# Intravenous methadone versus regional and neuraxial analgesic techniques in the peri‐operative period: a scoping review

**DOI:** 10.1002/anr3.70070

**Published:** 2026-06-17

**Authors:** Z. J. Lim, K. Rough, H. Dubey, S. Lee

**Affiliations:** ^1^ Department of Anaesthesia Eastern Health, Box Hill Hospital Box Hill VIC Australia; ^2^ Department of Anaesthesia Austin Health, Austin Hospital Heidelberg VIC Australia; ^3^ Department of Critical Care, Melbourne Medical School University of Melbourne Parkville VIC Australia; ^4^ Research Library Australian and New Zealand College of Anaesthetists Melbourne VIC Australia

**Keywords:** analgesia, intravenous methadone, neuraxial anaesthesia, opioid consumption, regional anaesthesia

## Abstract

There is a renewed interest in intravenous methadone as a long‐acting peri‐operative opioid. However, evidence comparing intravenous methadone with contemporary regional and neuraxial analgesic techniques remains unclear. This scoping review aimed to map the current evidence evaluating intravenous methadone against regional and neuraxial analgesic techniques in the peri‐operative period. This review conformed to Preferred Reporting Items for Systematic Reviews and Meta‐analyses (extension for Scoping Reviews) guidelines. Relevant articles were searched in Medline, Scopus, Web of Science, EMBASE and CENTRAL from January 2000 to May 2026. All studies were reviewed with no language restrictions. Four retrospective studies involving 1341 patients were included. Intravenous methadone was compared with intrathecal morphine, epidural analgesia and transversus abdominis plane blocks across cardiac, abdominal, thoracic, pancreatic and caesarean surgeries. Three studies reported higher postoperative pain scores among patients receiving intravenous methadone compared with neuraxial analgesia, although this did not translate to clinically significant differences in opioid consumption. Shorter vasopressor requirements and earlier mobilisation were reported in patients receiving intravenous methadone. Complications were inconsistently reported across studies. In conclusion, current evidence comparing intravenous methadone with regional and neuraxial analgesic techniques remains limited and highly heterogeneous. Although neuraxial techniques were associated with lower pain scores, this did not consistently translate into higher opioid consumption among patients receiving intravenous methadone. Prospective multicentre randomised controlled trials are required to better define the role of intravenous methadone within contemporary peri‐operative analgesic pathways.

## Introduction

Intravenous methadone is an opioid with a rapid onset but long elimination phase at higher doses [1]. This pharmacodynamic property has resulted in renewed interest in its role in managing postoperative pain by serving as a reliable, longer‐acting opioid [2]. Interest in intravenous methadone has cumulated with multiple systematic reviews and meta‐analyses evaluating its efficacy against short‐acting opioids [3]. However, evidence comparing intravenous methadone against contemporary regional anaesthetic techniques remains unclear. While regional anaesthesia reduces postoperative opioid consumption and improves functional outcomes, its use may be limited by technical challenges, patient refusal or by factors contraindicating its use [4]. The aim of this scoping review was to map the current evidence evaluating intravenous methadone use and regional anaesthetic techniques, including neuraxial anaesthesia. We hypothesise that compared with patients receiving regional anaesthetic techniques, patients receiving intravenous methadone will have clinically similar pain scores, satisfaction and functional recovery outcomes.

## Methods

This scoping review follows the recommended methodological framework [5]. The structure of this review is based on the Preferred Reporting Items for Systematic Reviews and Meta‐analyses (extension for Scoping Reviews) guidelines (PRISMA‐ScR) [6]. This review was conducted after the protocol was published on Open Science Framework (https://osf.io/95syh).

We included all studies with adult participants (≥ 18 years) which compared patients receiving intravenous methadone against patients receiving any kind of regional anaesthetic or neuraxial  procedure. No restrictions were placed based on type of surgery, type of regional anaesthetic, type of local anaesthesia or peri‐operative pathway.

Studies were included if intravenous methadone was prescribed to patients during the peri‐operative period and patients receiving regional anaesthesia for the same surgery were directly compared. Studies which did not compare intravenous methadone with any regional anaesthetic were excluded.

No specific outcomes were predefined. As this scoping review aimed to evaluate two distinct analgesic approaches, we expected studies to compare postoperative pain scores, patient satisfaction and functional recovery outcomes.

All published studies from 01/01/2000 to 05/05/2026 were included in this review. We excluded studies published before 2000 to reflect contemporary anaesthetic practice. Conference abstracts, case reports and case series were reviewed, but if no comparison was made, these were excluded. No language restrictions were applied.

Information sources included MEDLINE (Ovid), EMBASE (Ovid), Web of Science, Scopus and CENTRAL. The search strategy was conceptualised with a qualified medical librarian who specialised in anaesthesia and pain medicine. The full list of search terms is provided in Appendix S1. Search items were further refined following a review of 20 abstracts.

Where systematic reviews were identified, the included studies were analysed to include any articles which were not captured during our initial search. Trial protocols were investigated via their associated trial registration platform to identify pre‐prints or recent publications.

A review management programme (Covidence®, Melbourne, Australia) was used for abstract screening and full‐text review. A discussion about the aims of the scoping review occurred before formal abstract screening by two authors. A third author was available for adjudication, if inconsistencies in screening were observed. Full‐text review and structured data extraction were conducted for all included studies, with the reasons for exclusion reported.

As part of the full‐text data extraction, study type, methodology, interventions and comparator were collected. Specifically, methadone dosing, type of regional anaesthetic and local anaesthetic agents (and additives) were extracted. These findings were synthesised and reported in a narrative fashion, with congruent results presented as tables to present the available evidence. No formal risk of bias assessment was performed. Ethics approval was not required as only published data were collected.

## Results

**Figure 1:**
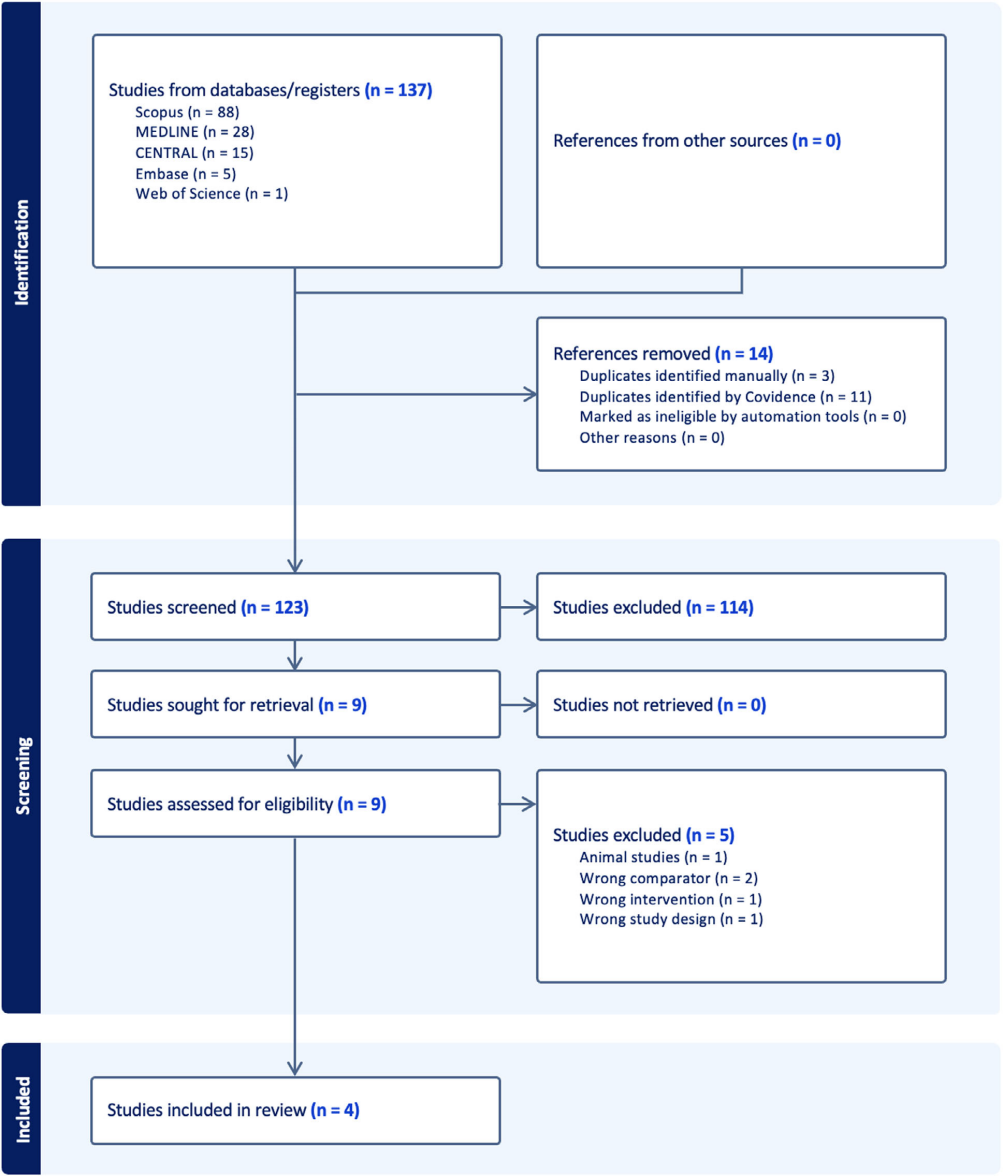
PRISMA‐ScR diagram.

The initial search conducted yielded 137 articles. Nine articles were selected for full‐text review, with five studies excluded. The study screening and selection process is summarised in Figure 1. The four studies included in this scoping review enrolled a total of 1341 patients [7, 8, 9, 10]. All studies were retrospective in nature. A summary of these studies is reported in Table 1 and outcomes in Table 2.

**Table 1:** Summary of selected studies.

Study	Study sample size	Surgery	Intervention	Comparison
	Country			
LaColla 2024 [7]	289 USA	Cardiac surgery	Intrathecal morphine 0.25 mg	Intravenous methadone 0.1 mg.kg^−1^
Pisters 2026 [8]	231 The Netherlands	Pancreatic resection	Epidural analgesia with bupivacaine 0.125% and sufentanil	Intravenous methadone 0.2 mg.kg^−1^
Rahrisch 2026 [9]	796 Switzerland	Laparotomy Non‐cardiac thoracotomy	Epidural analgesia with ropivacaine 0.2–0.3% and fentanyl	Intravenous methadone 0.2–0.3 mg.kg^−1^
Russell 2013 [10]	75 Australia	Caesarean birth	Transversus abdominis plane block	Intravenous methadone 0.17 ± 0.06 mg.kg^−1^

**Table 2:** Summary of outcomes.

Study	Pain score	Opioid consumption	Vasopressor use	Time to mobilisation
LaColla 2024 [7]	Pain scores between POD 0 to 3 were higher in IV‐M than in the ITM group.	No statistically significant difference reported.	Phenylephrine use was significantly higher in ITM group.	NR
Pisters 2026 [8]	Pain scores up to POD 2 were higher in IV‐M than in the epidural group.	No statistically significant difference reported.	Duration of vasopressor requirements was lower in IV‐M than in the epidural group.	Shorter in the IV‐M than in the epidural group (not statistically significant)
Rahrisch 2026 [9]	Pain scores up to POD 2 were higher in IV‐M than in the epidural group.	Lower morphine consumption (mean difference 6 mg) in IV‐M than in the epidural group.	Noradrenaline duration was longer in epidural group than in the IV‐M group.	Shorter in the IV‐M group (statistically significant)
Russell 2013 [10]	Pain scores up to POD 2 were lower in IV‐M than in the epidural group.	Lower morphine consumption in IV‐M than in the epidural group (total mean difference 98 mg)	NR	NR

POD, postoperative day; IV‐M, intravenous methadone; ITM, intrathecal morphine; NR, not reported.

Intravenous methadone was compared with neuraxial techniques in three studies [7, 8, 9]. Single‐shot intrathecal morphine was compared with intravenous methadone in one study analysing cardiac surgery patients [7]. Two studies compared intravenous methadone against epidural analgesia for laparotomies, pancreatic surgery and non‐cardiac thoracotomies [8, 9]. One study compared transversus abdominis plane (TAP) blocks with intravenous methadone for caesarean births under general anaesthesia [10]. Given the limited number of included studies, they are presented as a narrative synthesis below.

LaColla et al. compared intravenous methadone against intrathecal morphine in patients undergoing cardiac surgery across two medical institutions [7]. All patients were involved in the enhanced recovery after surgery protocol. The dose of single‐shot morphine was 0.25 mg (range 0.15–0.40 mg) and intravenous methadone was given at 0.1 mg.kg^−1^. Patients were given intravenous methadone if there were clear contraindications or patient refusal to intrathecal morphine.

Pisters et al. reported a single‐centre retrospective cohort study which compared epidural analgesia against intravenous methadone in both open and minimally invasive pancreatic surgery [8]. Patients who had epidural analgesia received 4–8 ml.h^−1^ of bupivacaine 0.125% and sufentanil 1 μg.ml^−1^, while intravenous methadone patients received 0.2 mg.kg^−1^ at induction of general anaesthesia.

Rahrisch et al. analysed patients who received either epidural analgesia or intravenous morphine for major abdominal and thoracic surgery [9]. Epidural analgesia with ropivacaine 0.3% was administered after induction of general anaesthesia, with the volume dependent on the patient's height. A continuous infusion of 5–8 ml.h^−1^ of ropivacaine 0.3% was administered throughout the surgery, followed by a continuous epidural infusion of 5–8 ml.h^−1^ of ropivacaine 0.2% plus 2 μg.ml^−1^ fentanyl. The patient could bolus 3 ml of this infusion per hour. In the intravenous methadone group, 0.2–0.3 mg.kg^−1^ based on ideal body weight was given after induction of general anaesthesia before skin incision.

Russell et al. compared the use of intravenous methadone against TAP blocks for patients undergoing general anaesthesia for caesarean birth [10]. Intravenous methadone was given at a mean dose of 0.17 mg.kg^−1^ (range 0.10–0.32 mg.kg^−1^) based on total body weight. The local anaesthetic and volume used for TAP blocks were not reported.

### Pain scores

Three out of four studies reported higher pain scores in patients receiving intravenous methadone compared to neuraxial analgesia [7, 8, 9]. When compared to intrathecal morphine use in cardiac surgery patients, pain scores were statistically significantly higher on postoperative days 0–3 in patients who received intravenous methadone, with the highest mean difference reported on postoperative day 0 (mean difference 0.95) [7]. Similarly, in non‐cardiac thoracotomies and laparotomies, pain scores were statistically higher in intravenous methadone patients compared to epidural analgesia (mean difference 0.42 over time) [9]. Among patients undergoing pancreatic surgery, multivariate analysis demonstrated that intravenous methadone patients had higher pain scores compared to epidural analgesia within the first 3 postoperative days [8].

In contrast, patients who received intravenous methadone undergoing general anaesthesia for caesarean births reported lower pain scores (0 versus 4.5 post‐anaesthesia; 3 versus 4 in 24 hours; 2 versus 2.5 in 48 hours) when compared to patients receiving TAP blocks [10].

### Opioid consumption

All four studies also reported on postoperative opioid consumption. Between intrathecal morphine and intravenous methadone, no significant difference in opioid consumption was reported from postoperative day 0 to 3 [7]. Among patients undergoing a laparotomy or non‐cardiac thoracotomy, no significant differences in postoperative opioid consumption were reported between intravenous methadone and epidural analgesia [9].

Reduced opioid consumption was observed when comparing intravenous methadone against epidural analgesia in patients undergoing pancreatic surgery (23 mg versus 29 mg morphine equivalent doses) [8]. Opioid consumption was also significantly lower in patients undergoing general anaesthesia for caesarean birth up to postoperative day 2 (115 mg versus 213 mg) [10].

### Vasopressor use

Three studies reported on postoperative vasopressor use. All studies reported significantly shorter duration of vasopressor use among patients receiving intravenous methadone compared to neuraxial analgesia [7, 8, 9].

### Time to mobilisation

Two studies reported on time to mobilisation. Both studies reported shorter time to mobilisation compared to neuraxial analgesia [8, 9], but only one study showed statistical significance [9].

### Complications

Complications following intravenous methadone were heterogeneously reported. Compared to neuraxial analgesia, intravenous methadone was associated with increased hospital length of stay among cardiac surgery patients [7]. However, the intravenous methadone group also had increased cardiopulmonary bypass and cross‐clamp time. Time to tracheal extubation among patients undergoing major abdominal or thoracic surgery was longer in the intravenous methadone group [9].

In contrast, intravenous methadone was associated with a shorter duration of urinary bladder catheterisation [8] and less need for laxatives [9].

Respiratory depression in the post‐anaesthesia care unit was reported in one study, in a patient who received 10 mg of methadone and 200 μg of fentanyl intra‐operatively after a general anaesthesia for caesarean birth [10].

## Discussion

This scoping review mapped the current evidence evaluating contemporary regional anaesthetic techniques against intravenous methadone for various surgeries. Although postoperative pain scores were higher in the intravenous methadone cohort, this did not translate to a higher postoperative opioid consumption. Intravenous methadone was associated with shorter vasopressor requirement and time to mobilisation. However, no randomised controlled trials were identified in the scoping review process, with all included studies reporting retrospective data.

The included studies highlight the potential role of intravenous methadone as a pragmatic alternative across diverse surgical populations when neuraxial analgesia is contraindicated or technically challenging. Although neuraxial analgesia remains an important component of peri‐operative pain management, its use may be limited by patient factors, such as anticoagulation, coagulopathy, infection risk, anatomical difficulty or refusal [11]. Additionally, neuraxial techniques may contribute to peri‐operative haemodynamic instability through sympathetic blockade [12]. In this context, this review presents intravenous methadone as a simpler and less invasive analgesic strategy which avoids the procedural limitations associated with neuraxial techniques.

An interesting observation from this scoping review was that higher postoperative pain scores in patients receiving intravenous methadone did not translate into increased opioid consumption. Although statistically significant differences in pain scores were reported in several studies, the observed mean differences were below the minimum clinically important difference [13]. In contrast, clinically meaningful reductions in postoperative consumption were observed in two studies comparing intravenous methadone with epidural analgesia and TAP blocks. The reasons underlying this observation are unclear. However, the prolonged duration of action and N‐methyl‐D‐aspartate receptor antagonism associated with methadone may contribute to opioid‐sparing effects in the postoperative period [14].

An additional consideration from this scoping review is the doses of intravenous methadone administered. The reported doses may have been lower than current doses proposed to optimise postoperative analgesia. Recent dose‐finding work suggests a single intra‐operative methadone dose of 0.25 mg.kg^−1^ of ideal body weight as the most favourable balance between its analgesic effects and adverse events. In this review, several studies may have administered doses below this optimal range, which may explain the higher postoperative pain scores observed [15].

Although further interpretations may be proposed from the available findings, this scoping review primarily highlights the paucity of evidence and substantial heterogeneity among studies evaluating intravenous methadone against regional and neuraxial analgesic techniques. Methadone dosing varied widely across studies, ranging from 0.1 to 0.3 mg.kg^−1^, while the comparator neuraxial regimes differed substantially in terms of local anaesthetic concentration, opioid adjuncts and infusion strategies. This variability limits any meaningful direct comparison between the selected studies. Furthermore, these limitations are further compounded by the absence of randomised controlled trials, increasing the risk of selection bias, confounding and institutional practice variation. Consequently, the current evidence remains insufficient to determine the comparative efficacy and safety of intravenous methadone relative to regional or neuraxial techniques across different surgical settings.

In conclusion, the current evidence comparing intravenous methadone with regional and neuraxial techniques remains limited and highly heterogeneous. Although lower pain scores were reported with neuraxial techniques, these differences did not translate into increased opioid consumption among patients receiving intravenous methadone. While intravenous methadone may represent a pragmatic alternative to regional techniques, the retrospective design of the included studies and inconsistent reporting of outcomes limit any definitive conclusion regarding efficacy and safety. Further prospective multicentre randomised controlled trials are required to define the role of intravenous methadone within contemporary peri‐operative analgesic pathways.

## Supporting information


**Appendix S1.** Full list of search terms.

**Data S1.** Preferred Reporting Items for Systematic Reviews and Meta‐Analyses extension for Scoping Reviews (PRISMA‐ScR) Checklist.
