# Supplementary material for: Intravenous methadone versus regional and neuraxial analgesic techniques in the peri‐operative period: a scoping review
Source: Anaesth Rep. 2026 Jun 17;14(1):e70070. doi: 10.1002/anr3.70070 (PMC13275201; doi:10.1002/anr3.70070)
Supplement: Supplementary file 1 — Appendix S1. Full list of search terms. [file ANR3-14-e70070-s001.docx]

**Appendix S1. Full list of search terms**

| **#** | **Searches** |
| --- | --- |
| 1 | (intravenous adj2 methadone).ti,ab. |
| 2 | (IV adj2 methadone).ti,ab. |
| 3 | ("I.V." adj2 methadone).ti,ab. |
| 4 | 1 or 2 or 3 |
| 5 | exp Methadone/ |
| 6 | methadone.ti,ab. |
| 7 | 5 or 6 |
| 8 | exp Administration, Intravenous/ |
| 9 | intravenous.ti,ab. |
| 10 | IV.ti,ab. |
| 11 | "I.V.".ti,ab. |
| 12 | 8 or 9 or 10 or 11 |
| 13 | 7 and 12 |
| 14 | exp Anesthesia, Conduction/ |
| 15 | exp Anesthesia, Epidural/ |
| 16 | exp Analgesia, Epidural/ |
| 17 | exp Anesthesia, Spinal/ |
| 18 | exp Anesthesia, Spinal/ |
| 19 | exp Injections, Epidural/ |
| 20 | exp Nerve Block/ |
| 21 | regional an?esth*.ti,ab. |
| 22 | conduction an?esth*.ti,ab. |
| 23 | neuraxial.ti,ab. |
| 24 | epidural*.ti,ab. |
| 25 | spinal an?esth*.ti,ab. |
| 26 | nerve block*.ti,ab. |
| 27 | (upper adj2 block*).ti,ab. |
| 28 | (lower adj2 block*).ti,ab. |
| 29 | 14 or 15 or 16 or 17 or 18 or 19 or 20 or 21 or 22 or 23 or 24 or 25 or 26 or 27 or 28 |
| 30 | exp Perioperative Medicine/ |
| 31 | exp Perioperative Period/ |
| 32 | exp Perioperative Care/ |
| 33 | exp Preoperative Care/ |
| 34 | exp Preoperative Period/ |
| 35 | exp Intraoperative Complications/ |
| 36 | exp Intraoperative Care/ |
| 37 | exp Intraoperative Period/ |
| 38 | exp Postoperative Complications/ |
| 39 | exp Postoperative Period/ |
| 40 | exp Postoperative Care/ |
| 41 | (perioperat* or peri-operat*).ti,ab. |
| 42 | (preoperat* or pre-operat*).ti,ab. |
| 43 | (intraoperat* or intra-operat*).ti,ab. |
| 44 | (postoperat* or post-operat*).ti,ab. |
| 45 | 30 or 31 or 32 or 33 or 34 or 35 or 36 or 37 or 38 or 39 or 40 or 41 or 42 or 43 or 44 |
| 46 | 4 and 29 and 45 |
| 47 | limit 46 to yr="2000 -Current" |
| 48 | from 47 keep 5-6, 12-13 |
| 49 | 13 and 29 and 45 |
| 50 | limit 49 to yr="2000 -Current" |
| 51 | 50 not 47 |
| 52 | from 51 keep 18 |
| 53 | 4 and 29 |
| 54 | limit 53 to yr="2000 -Current" |
| 55 | 54 not 47 |
| 56 | 55 not 51 |
| 57 | 13 and 29 |
| 58 | limit 57 to yr="2000 -Current" |
| 59 | 58 not 47 |
| 60 | 59 not 51 |
| 61 | 60 not 56 |
| 62 | 4 and 45 |
| 63 | limit 62 to yr="2000 -Current" |
| 64 | 63 not 47 |
| 65 | 64 not 51 |
| 66 | 65 not 56 |
| 67 | 66 not 61 |
| 68 | from 67 keep 2-4, 6, 12, 16, 20, 32... |
| 69 | 13 and 45 |
| 70 | limit 69 to yr="2000 -Current" |
| 71 | 70 not 47 |
| 72 | 71 not 51 |
| 73 | 72 not 56 |
| 74 | 73 not 61 |
| 75 | 74 not 67 |
| 76 | from 75 keep 2, 17, 26, 37, 44, 48… |
| 77 | limit 4 to yr="2000 -Current" |
| 78 | 77 not 47 |
| 79 | 78 not 51 |
| 80 | 79 not 56 |
| 81 | 80 not 61 |
| 82 | 81 not 67 |
| 83 | 82 not 75 |
| 84 | 48 or 52 or 68 or 76 |
